# Genomics Analysis of L-DOPA Exposure in *Drosophila sechellia*

**DOI:** 10.1534/g3.119.400552

**Published:** 2019-10-01

**Authors:** Stephen M. Lanno, Ivy Lam, Zachary Drum, Samuel C. Linde, Sara M. Gregory, Serena J. Shimshak, Mariel V. Becker, Kerry E. Brew, Aashli Budhiraja, Eliza A. Carter, Lorencia Chigweshe, Keagan P. Collins, Timothy Earley, Hannah L. Einstein, Angela A. Fan, Sarah S. Goss, Eric R. Hagen, Sarah B. Hutcheon, Timothy T. Kim, Mackenzie A. Mitchell, Nola R. Neri, Sean E. Patterson, Gregory Ransom, Guadalupe J. Sanchez, Bella M. Wiener, Dacheng Zhao, Joseph D. Coolon

**Affiliations:** Department of Biology, Wesleyan University, Middletown, CT 06457

**Keywords:** L-DOPA, oogenesis, host specialization, adaptation, RNA-seq, esterase

## Abstract

*Drosophila sechellia* is a dietary specialist fruit fly that evolved from a generalist ancestor to specialize on the toxic fruit of *Morinda citrifolia*. This species pair has been the subject of numerous studies where the goal has largely been to determine the genetic basis of adaptations associated with host specialization. Because one of the most striking features of *M. citrifolia* fruit is the production of toxic volatile compounds that kill insects, most genomic studies in *D. sechellia* to date have focused on gene expression responses to the toxic compounds in its food. In this study, we aim to identify new genes important for host specialization by profiling gene expression response to 3,4-dihydroxyphenylalanine (L-DOPA). Recent work found it to be highly abundant in *M. citrifolia*, critical for reproductive success of *D. sechellia*, and supplementation of diet with the downstream pathway product dopamine can influence toxin resistance phenotypes in related species. Here we used a combination of functional genetics and genomics techniques to identify new genes that are important for *D. sechellia* ecological adaptation to this new niche. We show that L-DOPA exposure can affect toxin resistance phenotypes, identify genes with plastic responses to L-DOPA exposure, and functionally test an identified candidate gene. We found that knock-down of *Esterase 6* (*Est6*) in a heterologous species alters toxin resistance suggesting *Est6* may play an important role in *D. sechellia* host specialization.

In insects, the complex nature of plant-insect interactions, multi-trophic interactions involving predator and prey dynamics, and indirect effects between and among them contribute to adaptations. Most plant-feeding insects are dietary specialists that feed on a small number of closely-related plant species (Price *et al.* 1980; Jaenike 1990; Bernays and Chapman 1994). Often, this specialization is the result of host-specific adaptations due to variation in plant chemistry. However, determining the specific genetic changes that accompany specialization or host shifts remains challenging. *Drosophila sechellia*, a dietary specialist fruit fly endemic to the Seychelles Islands (Tsacas and Bachli 1981), is a well-suited model system for examining questions related to adaptive host specialization. *D. sechellia* has evolved to specialize its feeding, metabolism, oviposition, and development on the toxic fruit of *Morinda citrifolia* (Louis and David 1986; Matute and Ayroles 2014). Its close phylogenetic relationship with the model organism *D. melanogaster* (divergence time ∼3 MYA, Clark *et al.* 2007) and sister species *D. simulans* (divergence time <100 KYA, Schrider *et al.* 2018), both dietary generalists, gives *D. sechellia* exceptional power for dissecting the underlying genetics involved in host specialization and evolved resistance to plant defense compounds. Toxicity of *M. citrifolia* fruit is predominantly due to high levels of the medium chain fatty acid octanoic acid (OA) which *D. sechellia* has evolved resistance to and preference for (Legal *et al.* 1992, 1994; Farine *et al.* 1996). Many studies have investigated the genetic basis of toxin resistance in *D. sechellia* (R’Kha *et al.* 1991; Amlou *et al.* 1998a,b; Jones 1998, 2001; Hungate *et al.* 2013; Huang & Erezyilmaz 2015; Andrade López *et al.* 2017; Lanno *et al.* 2017; Peyser *et al.* 2017; Lanno *et al.* 2019), where most focus solely on the highly abundant and toxic compound OA. However, it is possible that an interaction or synergistic effect of other compounds found in the fruit contributes to both lethality and resistance.

In addition to being toxic to other insects, *M. citrifolia* fruit also contains high levels of 3,4-dihydroxyphenylalanine (L-DOPA), a chemical precursor of dopamine. Tyrosine Hydroxylase (TH), encoded by the pale (*ple*) locus, mediates the conversion of tyrosine to L-DOPA (Nagatsu *et al.* 1964; Budnik and White 1987; Neckameyer and White 1993). Homozygous null alleles of this gene as well as pharmacological inhibition of TH result in embryonic lethality (Neckameyer and White 1993; Neckameyer 1996; Pendleton *et al.* 1996). Alongside TH, a group of genes surrounding the Dopa Decarboxylase (*Ddc*) locus, which catalyzes the decarboxylation of L-DOPA to dopamine, also play an important role in catecholamine metabolism (Wright 1987; Stathakis *et al.* 1995). Of particular interest is Catecholamines up (*Catsup*), a gene within this group that encodes a protein that regulates TH activity that was discovered to contain loss of function mutations in *D. sechellia*, driving this species to become reliant on its obligate host, *M. citrifolia* (Stathakis *et al.* 1999; Lavista-Llanos *et al.* 2014). In contrast to other *Drosophila* species, *D. sechellia* has much lower levels of cellular L-DOPA while still maintaining high levels of dopamine. This is achieved by consuming *M. citrifolia* that produces large amounts of L-DOPA in its fruit. L-DOPA plays several roles in plants, including inhibiting growth of competing plant species, as well as acting as a secondary defense compound in some cases (Soares *et al.* 2014). Lavista-Llanos *et al.* (2014) found that supplementing food with dopamine increased *D. melanogaster* resistance to *M. citrifolia* fruit toxins (Lavista-Llanos *et al.* 2014). However, whether the high levels of L-DOPA naturally co-occurring in *Morinda* fruit (and not dopamine) contributes to OA resistance remains unknown.

Here we functionally test the role of L-DOPA in OA resistance by performing mortality assays in the presence of L-DOPA, perform RNA-sequencing on control *vs.* L-DOPA exposed flies to identify candidate genes that may be involved in L-DOPA mediated toxin resistance, and functionally test an identified candidate for effects on OA resistance.

## Materials and Methods

### Fly strains and culture

*Drosophila sechellia* (14021-0428.25), *D. simulans* (14021-0251.195), and *D. melanogaster* (14021-0231.36, BDSC:55927 (EST6 RNAi), GeneSwitch-GAL4 line (*Tubulin*-P[Switch]) flies were reared on cornmeal medium using a 16:8 light:dark cycle at 20°. Adult females of each species were collected at 0-3 days post-eclosion and exposed to either control food (0.75g *Drosophila* instant medium Equation 4-24, Carolina Biological Supply Company) or food containing L-DOPA (10mg/ml, concentration chosen from observations of dopamine supplementation described in Lavista-Llanos *et al.* 2014) for 24 hr. After exposure, flies were either used in mortality assays or for measurement of genome-wide gene expression.

### Mortality assay

The mortality assays used in this work were performed according to methods described in prior studies (Andrade López *et al.* 2017; Peyser *et al.* 2017; Lanno & Coolon 2019; Lanno *et al.* 2019). Briefly, control and L-DOPA exposed flies of all three species were transferred into vials (10 per vial) containing 0.75g *Drosophila* medium supplemented with 1.2% octanoic acid (OA, Sigma) (Andrade López *et al.* 2017; Peyser *et al.* 2017; Lanno & Coolon 2019; Lanno *et al.* 2019). Flies used for mortality assays were all 1-4 day old females for each species and each sample type was collected in replicate six-eight times with 10 flies per replicate (N = 60 to 80 per sample type depending on the experiment, see below). OA resistance was measured by determining the number of flies ‘knocked down’ (a fly was determined to be knocked down when it was no longer able to walk or fly) every 5 min for a period of 60 min.

### RNA interference

RNA interference (RNAi) was performed to knockdown the expression of *Esterase 6* by first crossing the UAS-Est6-RNAi line that expresses a hairpin RNA under the control of UAS to GeneSwitch-GAL4 which expresses a modified chimeric GAL4 ubiquitously that will only become active in the presence of the synthetic antiprogestin mifepristone (RU486). Therefore, RNAi targeting *Est6* will only occur in individuals that have both UAS-Est6-RNAi and GeneSwitch-GAL4 in the presence of RU486 (Osterwalder *et al.* 2001; Roman *et al.* 2001; Andrade López *et al.* 2017; Lanno *et al.* 2019). Adult female progeny from this cross were collected at 0-3 days post-eclosion and treated with either 10µg/ml RU486 (knockdown) or 10µl/ml EtOH (control) added directly to their media for 24 hr. After exposure, flies were used in mortality assays which also contained RU486 to maintain knockdown in those samples.

### Cox proportional hazards regression analysis

A Cox proportional hazards statistical model was used to test the effect of L-DOPA exposure on OA associated mortality using the *coxph* command in the *survival* package in R (Cox 1972; Fox 2008; Therneau 2015; Andrade López *et al.* 2017; Peyser *et al.* 2017; R Core Development Team 2017; Lanno & Coolon 2019; Lanno *et al.* 2019). We report relative survival as the regression coefficient (-β) for each treatment group compared to its species-specific control group (with *vs.* without L-DOPA). Sample size for the effect of L-DOPA on OA resistance was N = 60 per treatment. Blocking by vial was included in the model and found to have no effect. In the RNAi experiment where we compare RNAi knockdown to uninduced controls (with *vs.* without knockdown) we used a separate Cox model to test for the effect of knockdown of *Est6* on OA resistance and the sample size was N = 80 for each treatment. Blocking by vial was again included in the model and found to have no effect.

### RNA extraction, library preparation and RNA- sequencing

After exposure to the control or L-DOPA food sources, flies of each species were flash frozen in liquid nitrogen and kept at -80° until RNA extraction. The Promega SV total RNA extraction system with modified protocol (Coolon *et al.* 2012) was used to extract RNA from a homogenate of 10 whole adult female flies per replicate per species per treatment. Three biological replicates were analyzed for each species and exposure environment for a total of 18 sequencing libraries (Table 1). Prior to library preparation, NanoDrop and subsequent gel electrophoresis were used to determine the quantity and quality of RNA extracted. All RNA samples were sent to the University of Michigan Medical School DNA Sequencing Core Facility for mRNA selected library preparation and sequencing. Bar-coded sequencing libraries were made using TruSeq library preparation kits and pooled for sequencing. Uniform library representation of each library was confirmed with qPCR prior to sequencing. The pooled barcoded libraries were sequenced on two lanes of an Illumina HiSeq-4000 generating single end sequence reads for subsequent analyses.

**Table 1:** Percent mapped reads for sequencing libraries

Sample	# Reads	# Mapped Reads	% Mapped
*D. sechellia* C1	19,222,060	18,496,450	96.23
*D. sechellia* C2	20,704,811	19,440,620	93.89
*D. sechellia* C3	17,696,868	17,123,579	96.76
*D. sechellia* LD1	19,576,162	18,341,777	93.69
*D. sechellia* LD2	14,508,205	12,988,684	89.53
*D. sechellia* LD3	17,432,600	16,040,372	92.01
*D. simulans* C1	28,056,123	26,210,691	93.42
*D. simulans* C2	26,058,213	24,449,785	93.83
*D. simulans* C3	24,095,284	22,589,715	93.75
*D. simulans* LD1	17,841,650	16,739,731	93.82
*D. simulans* LD2	14,608,378	13,562,252	92.84
*D. simulans* LD3	17,628,201	16,471,452	93.44
*D. melanogaster* C1	21,999,530	20,633,866	93.79
*D. melanogaster* C2	20,950,464	19,779,953	94.41
*D. melanogaster* C3	22,157,160	20,919,514	94.41
*D. melanogaster* LD1	39,619,560	37,618,491	94.95
*D. melanogaster* LD2	23,214,861	21,991,775	94.73
*D. melanogaster* LD3	18,976,326	17,855,382	94.09

### BIOL310 Genomics Analysis

The genomics analysis of RNA-seq data presented in this manuscript was performed by 18 undergraduate and 2 graduate students as part of a semester-long course at Wesleyan University called Genomics Analysis (BIOL310). This is the second such manuscript (see Lanno *et al.* 2017) made from this course where the aim is to provide undergraduate students with a course-based research experience where they actively participate in the process of scientific discovery. The students learn through engaging with never-before analyzed data where they learn how to use cutting edge genomics analysis techniques and bioinformatics tools through a discovery-based independent study. Each student in the course contributed to the analyses and write-up of the findings, providing their own unique interpretation of the results and text written by each and every student was combined into this manuscript.

After sequence reads were returned by the University of Michigan Sequencing Core (Table 1), an RNA-seq analysis pipeline was performed in the online Galaxy environment (https://usegalaxy.org/, Afgan *et al.* 2016). Sequencing output files for each sample were quality control checked using FASTQC (Andrews 2010) and identified overrepresented sequences were identified using NCBI BLAST (Altschul *et al.* 1990). Sequence reads were mapped to the corresponding species genome using Bowtie2 with default parameters (Langmead and Salzberg 2012). The most current genome files at the time of analysis were obtained from Ensembl (Yates *et al.* 2016) (*D. sechellia*: Drosophila_sechellia.dsec_caf1.dna.toplevel.fa, *D. simulans*: Drosophila_simulans.ASM75419v3.dna.toplevel.fa and *D. melanogaster*: Drosophila_melanogaster.BDGP6.dna.toplevel.fa). Quantification of gene expression and differential expression tests were performed with Cuffdiff (Trapnell *et al.* 2010, 2013) using the genome files described above and the gene annotation files available from Ensembl at the time of analysis (*D. sechellia*: Drosophila_sechellia.dsec_caf1.42.gff3, *D. simulans*: Drosophila_simulans.ASM75419v3.42.gff3 and *D. melanogaster*: Drosophila_melanogaster.BDGP6.95.gff3). Geometric normalization and gene length correction options in Cuffdiff were used to improve comparisons of gene expression. False discovery rate multiple testing correction (Benjamini and Hochberg 1995) was used to account for the multitude of simultaneously conducted tests. Data visualization and processing was performed in R (R Core Development Team 2017). In order to compare gene expression results across species, we obtained all 1:1:1 orthologs from *D. sechellia*, *D. simulans* and *D. melanogaster* from Flybase (Attrill *et al.* 2016). We performed Gene Ontology enrichment analysis with the Gene Ontology Consortium online tool (http://geneontology.org/, Ashburner *et al.* 2000; Blake *et al.* 2015) using the annotations from the *D. melanogaster* orthologs.

### DNA coding and protein sequence analyses of Est6

DNA coding sequences (CDS) for *Est6* were downloaded from FlyBase (Attrill *et al.* 2016) for *D. melanogaster*, *D. sechellia*, and *D. simulans*. Clustal Omega (Goujon *et al.* 2010; Sievers *et al.* 2011; McWilliam *et al.* 2013) was used to align DNA CDS and translated protein sequences in order to determine synonymous and nonsynonymous differences between these species. To investigate *Est6* CDS variation in multiple *D. sechellia* genotypes, paired-end DNA sequencing files from 23 wild-caught *D. sechellia* genomes from the Seychelles islands were downloaded from NCBI’s Short Read Archive (BioProject number PRJNA395473) (Schrider *et al.* 2018). Each file was mapped to a fasta file containing the *D. sechellia Est6* DNA CDS using Bowtie 2 (Langmead and Salzberg 2012). Aligned reads were then assessed for variation among *D. sechellia* lines for the *Est6* allele using the Naïve Variant Caller (Blankenberg *et al.* 2014) in Galaxy using the *D. sechellia Est6* DNA CDS downloaded from FlyBase as a reference (Attrill *et al.* 2016).

### Data accessibility

All RNA-seq data generated in this manuscript have been submitted to the NCBI Gene Expression Omnibus under accession number GSE138119. Supplemental material available at figshare: https://doi.org/10.25387/g3.8938103.

## Results

### Testing species-specific L-DOPA mediated resistance to octanoic acid

**Figure 1:**
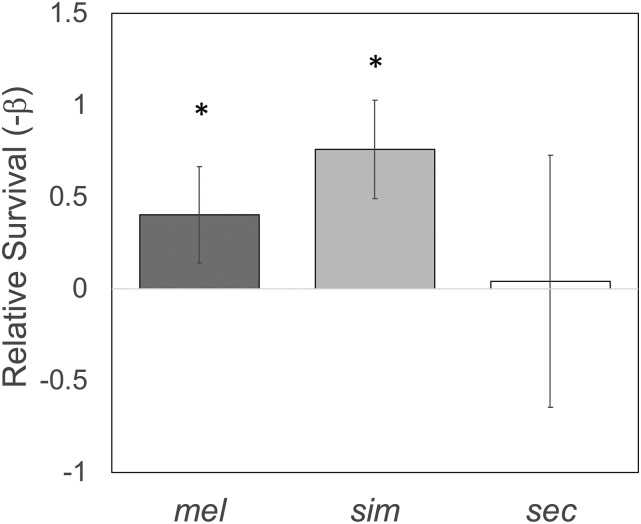
L-DOPA effect on OA resistance. After 24 hr of exposure to 10mg/ml L-DOPA, *D. melanogaster*, *D. simulans* and *D. sechellia* adult flies were tested for changes in OA resistance. Plotted are relative survival (-β) estimates comparing each species OA resistance with and without exposure to L-DOPA. Askterisk indicates significant effect of L-DOPA on OA resistance in that species (*P* < 0.05).

Prior work showed that *D. melanogaster* strains had increased resistance to a combination of octanoic and hexanoic acids when concurrently given dopamine in their media (Lavista-Llanos *et al.* 2014). However, *M. citrifolia* produces high levels of L-DOPA and not dopamine and the consequences of L-DOPA exposure on toxin resistance remain unknown. Furthermore, because *D. sechellia* was also not included in this test, it is unknown how *D. sechellia* responds to L-DOPA in their diet and possible effects on toxin resistance. To test for L-DOPA mediated toxin resistance, we performed octanoic acid (OA) resistance assays with and without L-DOPA supplementation using three closely-related species: *D. sechellia*, *D. simulans* and *D. melanogaster*. Flies were fed media containing 10mg/ml L-DOPA for 24 hr prior to measurement of OA resistance. We found that L-DOPA supplementation significantly increased both *D. melanogaster* (Cox Proportional Hazards Test, -β =1.06, *P* = 3.1 **×** 10^−7^) and *D. simulans* (Cox Proportional Hazards Test, -β =0.99, *P* = 3.1 **×** 10^−7^) resistance to OA, but there was no effect on *D. sechellia* (Cox Proportional Hazards Test, -β=0.63, *P* = 0.11) (Figure 1). Because *D. sechellia* is so highly resistant to OA (only 2/120 died in the experiment in total), our test using a concentration of OA consistent with the maximum biologically available OA in *M. citrifolia* fruit, representing a reasonable natural condition is not capable of identifying an increase (not statistically possible) in OA resistance for this species in response to L-DOPA. Therefore, it remains unknown whether L-DOPA influences OA resistance in *D. sechellia*.

### Investigating Drosophila gene expression responses to dietary L-DOPA

**Figure 2:**
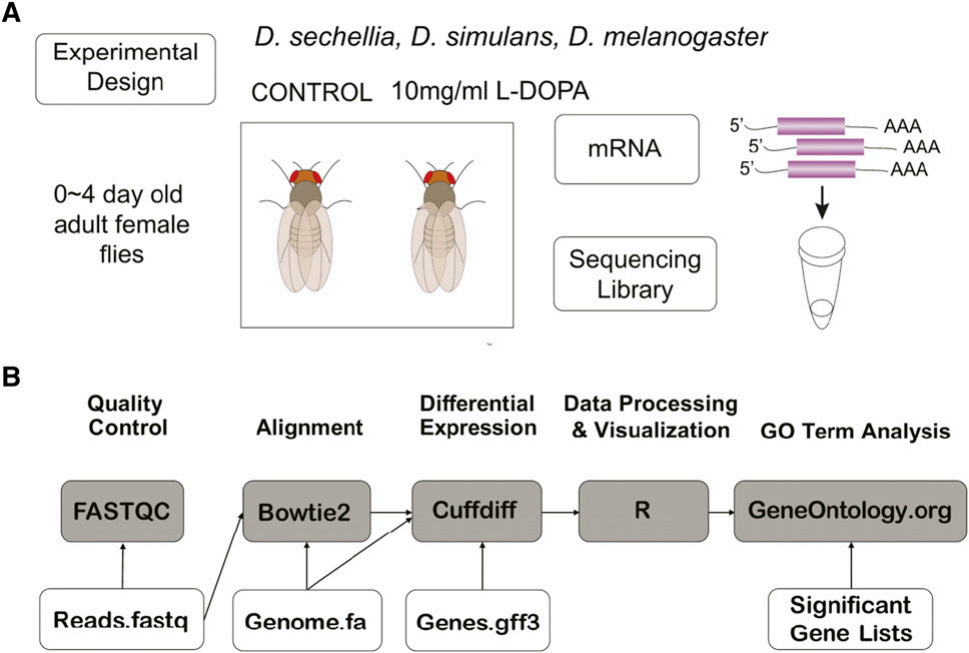
RNA-seq experimental design. A) Adult female flies were fed either control food or food mixed with 10 mg/ml L-DOPA for 24 hr followed by RNA extraction, Illumina library preparation and sequencing on an Illumina Hiseq 4000. B) Data generated by the Illumina sequencing were analyzed using the bioinformatics pipeline implemented on the Galaxy platform (usegalaxy.org). The process included quality control with FASTQC, alignment with Bowtie2, normalization and differential expression testing with Cuffdiff, processing and visualization in R, and gene ontology (GO) term enrichment tests performed at GeneOntology.org (Ashburner *et al.* 2000; Blake *et al.* 2015).

We next sought to identify gene expression responses of all three species to L-DOPA exposure with the goal of identifying candidate genes that may play a role in host specialization. Prior work has shown that genes whose expression is responsive to environmental conditions are important for fitness in those environments (Coolon *et al.* 2009; Lanno *et al.* 2017). Furthermore, Lavista-Llanos found that not only is toxin resistance altered by exposure to dopamine, but various aspects of egg production as well (Lavista-Llanos *et al.* 2014). In order to identify candidate genes that may play a role in OA resistance and egg production in the presence of L-DOPA exposure, we used RNA-seq to measure genome-wide gene expression in adult female *D. melanogaster*, *D. simulans* and *D. sechellia* with and without L-DOPA supplemented in their media (Figure 2).

**Figure 3:**
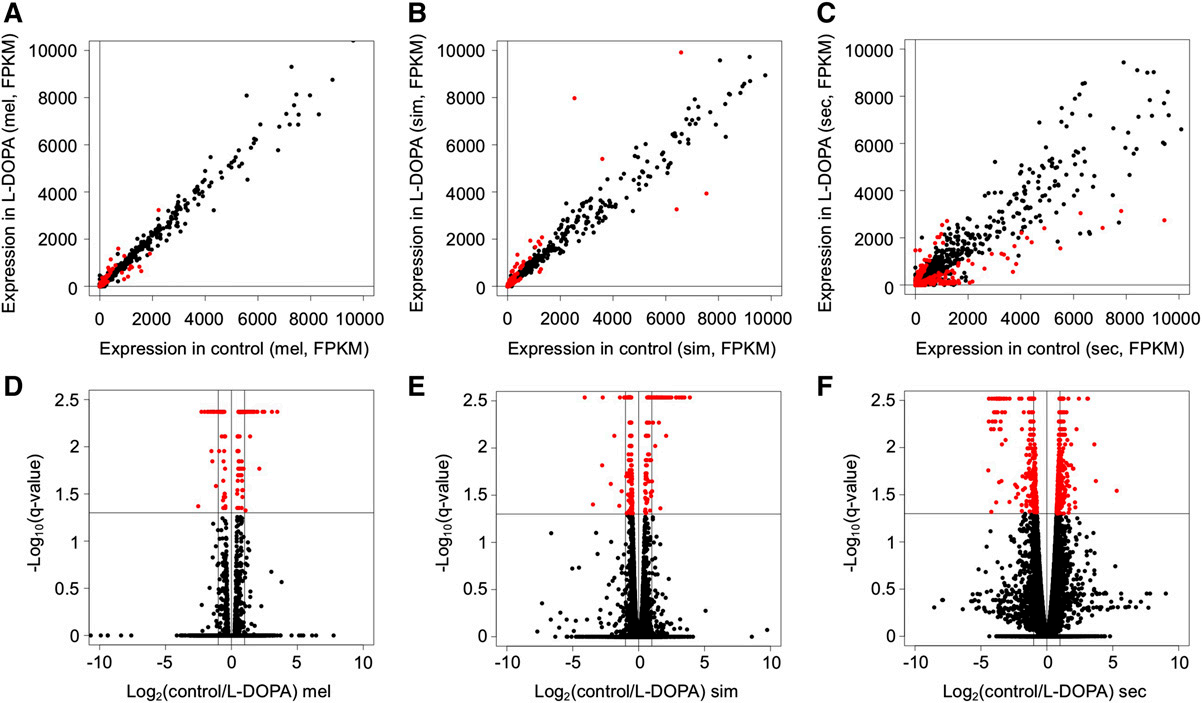
Genome-wide gene expression responses to L-DOPA. A-C) Scatterplots are shown for the comparison between flies fed control food (X-axis) and those fed food containing 10µg/ml L-DOPA (y-axis) with gene expression represented as F ragments P er K ilobase of sequence per M illion reads (FPKM) where each point is one gene with A) *D. melanogaster*, B) *D. simulans* and C) *D. sechellia*. D-F) Volcano plots are shown for the same comparison (control *vs.* 10µg/ml L-DOPA) for each species D) *D. melanogaster*, E) *D. simulans* and F) *D. sechellia* where expression response represented as Log(control FPKM/L-DOPA FPKM) on the x-axis and the significance of a statistical test for that gene represented as -Log_10_(q-value) where q is the false discovery rate corrected p-value on the y-axis.

**Figure 4:**
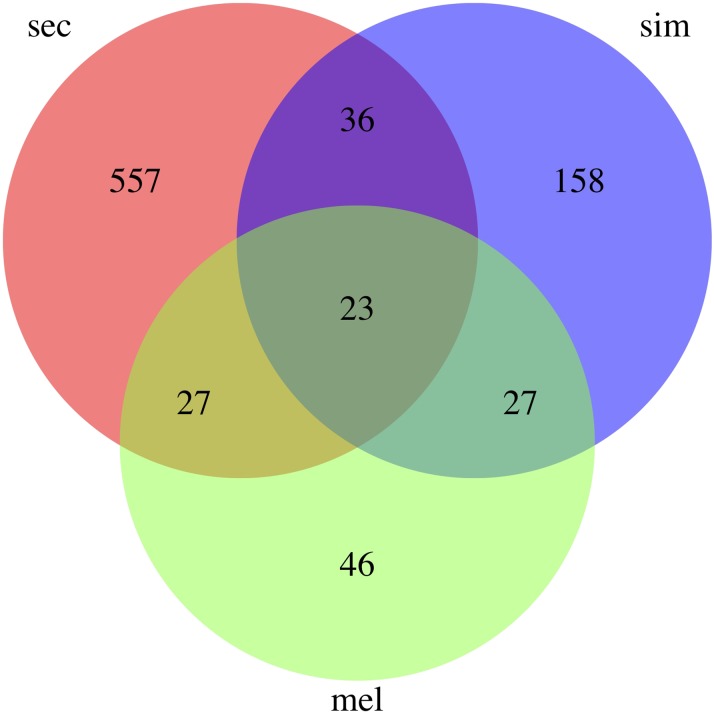
Venn diagram of L-DOPA responsive genes. The intersection of the genes identified by each of the three RNA-seq comparisons of flies fed control food *vs.* food supplemented with 10mg/ml L-DOPA are shown.

RNA-seq libraries were sequenced yielding a total of 384,346,456 sequence reads for the project and an average of 21 million per library (Table 1). FASTQC analysis (Andrews 2010) of the reads showed that they were high-quality and no trimming or sequence filtering was necessary prior to downstream analysis. We aligned the sequence reads to the corresponding genome with Bowtie2 (Langmead and Salzberg 2012) (see methods) and 94% of sequences on average aligned uniquely per library to the corresponding genome (Table 1). These alignments were then used for quantification of gene expression and differential expression testing with Cuffdiff (Trapnell *et al.* 2010, 2013). We found that 123 genes were significantly differentially expressed by *D. melanogaster* (Figure 3A,D, Supplemental Table 1), 244 by *D. simulans* (Figure 3B,E, Supplemental Table 2) and 643 by *D. sechellia* (Figure 3C,F, Supplemental Table 3) in response to dietary L-DOPA. Interestingly, the largest number was differentially expressed by *D. sechellia*, the only species that routinely has high concentrations of L-DOPA in its natural food source. There were 557 genes that were only plastic in response to L-DOPA by *D. sechellia* and not identified as differentially expressed by *D. melanogaster* or *D. simulans* (Figure 4, Supplemental Table 4), representing an interesting set of genes for further analysis because these genes may mediate L-DOPA associated phenotypic plasticity (*e.g.*, increased egg production, Lavista-Llanos *et al.* 2014). Finally, there were 23 genes differentially expressed by all three species that are interesting for their conserved gene expression response across species (Supplemental Table 5).

### Gene ontology term enrichment analysis

In order to determine if there is enrichment for genes with particular functions among those differentially expressed in response to L-DOPA, we tested the sets of significantly upregulated and downregulated genes identified from each species for gene ontology (GO) term enrichment. We found that there were very few enriched terms for upregulated and downregulated genes from *D. melanogaster* or *D. simulans* (Supplemental Tables 6-9). There were no enriched GO terms for upregulated genes from *D. melanogaster* or *D. simulans*. There were only two enriched GO terms for in downregulated genes in *D. melanogaster*, chorion (GO:0042600) and external encapsulating structure (GO:0030312) (Supplemental Table 6). There were multiple enriched terms for *D. simulans* downregulated genes associated with cellular parts that were non informative (Supplemental Table 7) and there was enrichment for phosphoric ester hydrolase activity (GO:0042578). Finally, there were numerous functions enriched in both the up and down-regulated genes in *D. sechellia* with far more terms enriched in the upregulated gene set (Supplemental Table 8). The enriched functions from downregulated genes are predominantly associated with various classes of genes with peptidase activity (Supplemental Table 8). The numerous terms enriched for upregulated genes in *D. sechellia* include multiple terms associated with increased reproductive output including mitosis/meiosis, cell cycle, DNA replication, gamete generation (Supplemental Table 8). We next investigated GO term enrichment in the set of 23 genes found in all 3 comparisons and found that the majority of terms enriched had to do with egg production including aspects of vitelline membrane formation, egg coat formation, chorion and extracellular matrix formation (Supplemental Table 9).

### Testing the function of a candidate gene in OA resistance

**Figure 5:**
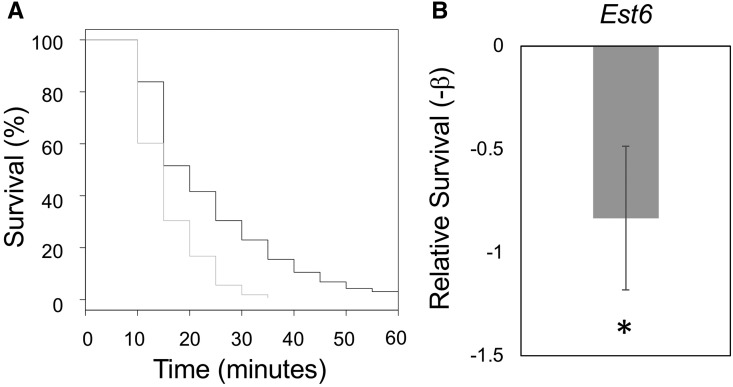
Knockdown of *Est6* causes reduction in OA resistance. After 24 hr of exposure to RU486, *D. melanogaster* GeneSwitch-GAL4 × UAS-EST6-RNAi adults female flies were tested for changes in OA resistance. Percent survival across time is plotted in A and relative survival (-β) estimates comparing individuals with and without exposure to RU486 and measuring OA resistance are shown in B. * indicates significant *Est6* knockdown effect on OA (*P* < 0.05).

Many of the genes identified as responding to L-DOPA exposure are excellent candidates for various aspects of host specialization in *D. sechellia* including possible roles in reproduction (Lavista-Llanos *et al.* 2014), mate recognition/reproductive isolation (*e.g.*, *EloF*, Supplemental Table 4, Combs *et al.* 2018), behavioral changes (Yamamoto and Seto 2014), cuticle formation (Lanno *et al.* 2017) and toxin resistance (Lavista-Llanos *et al.* 2014). For example, we found that *D. sechellia* response to L-DOPA includes marked increase in the expression of *Esterase 6* (*Est6*, Supplemental Table 4). In our recent study, we found that one or more esterase genes are involved in *D. sechellia* derived resistance to OA through experiments with the chemical synergist tribufos (S,S,S-Tributyltrithiophosphate) that inhibits all the esterase genes simultaneously (Lanno and Coolon 2019). To determine if *Est6* plays a role in evolved OA resistance we used RNAi in *D. melanogaster* to reduce the expression of *Est6* and examined the effect on OA resistance. Using the ubiquitously expressed GeneSwitch-GAL4 crossed into UAS-EST6-RNAi line yields individuals that are wildtype unless they are exposed to the synthetic antiprogestin mifepristone (RU486), which induced RNAi knockdown of *Est6*. We compared genotypically identical siblings with and without RU486 exposure and found that knockdown of *Est6* caused a significant decrease in resistance to OA (Cox Proportional Hazards Test, -β = -0.833, *P* = 1.7 **×** 10^−7^, Figure 5). To determine whether altered protein sequence could also contribute to *Est6* functional differences in addition to the observed gene expression responses, we compared the protein sequences for *Est6* from *D. melanogaster*, *D. simulans* and *D. sechellia* and identified 5 derived amino acids in *D. sechellia*. One of the derived residues (H187A) affects an amino acid that resides in the active site of the enzyme (Younus *et al.* 2017) that was previously shown to influence *Est6* substrate specificity and enzyme kinetics (the specific amino acid substitution found in *D. sechellia* was not tested but other residue substitutions at this position had the stated effects (Myers *et al.* 1993). Using available genomes from several *D. sechellia* isolates (Schrider *et al.* 2018) we have confirmed that the mutations responsible for this amino acid substitution are present in all *D. sechellia* sequenced to date suggesting this a fixed sequence difference in this species.

## Discussion

Here we found that adult *D. melanogaster* and *D. simulans* exposed to L-DOPA have increased resistance to OA. We do not see this effect for *D. sechellia* because *D. sechellia* is so highly resistant to OA that our test could not test for increased resistance. In an effort to maintain ecological relevance of the results we have used a concentration of OA that best matches the maximum biologically available OA in *M. citrifolia* fruit. As such, it remains unknown whether L-DOPA influences OA resistance in *D. sechellia*. To identify genes that might contribute to host specialization downstream of L-DOPA exposure, we used RNA-seq, to determine the genes with expression plasticity in response to L-DOPA, with a specific interest in those that had derived plasticity in *D. sechellia* because they may represent good candidates for genes involved in *D. sechellia* specialization on *M. citrifolia* that contains high concentrations of L-DOPA in its fruit.

### Identification of candidate genes

Included in the genes identified by the RNA-seq analysis were several candidates that may play a role in different aspects of *D. sechellia* host specialization. Specifically, we focused on genes with evolved transcriptional responses to L-DOPA in *D. sechellia* that were not identified as responsive to L-DOPA in *D. melanogaster or D. simulans* (Supplemental Table 4). Among these genes were several that have been identified in prior studies as possible contributors to different aspects of evolutionary changes in *D. sechellia*. For example, the fatty acid elongase *eloF* was shown to influence the abundance of different length fatty acids found on female flies with fewer longer cuticular hydrocarbon (CHC) species observed when the gene was knocked out (Combs *et al.* 2018). Interestingly, *D. simulans* flies are quite similar to the *D. sechellia **eloF* knockout flies in CHC profiles suggesting that this may be a mechanism of species discrimination that influences mate choice. When *eloF* was knocked out in *D. sechellia* female flies, male *D. simulans* individuals courted them as if they were *D. simulans* females demonstrating the role *eloF* has played in reproductive isolation between these species (Combs *et al.* 2018). Here we found that *D. sechellia* significantly decreases expression of *eloF* in the presence of L-DOPA (Supplemental Table 4), something only observed in this species. This species-specific reduction in expression could suggest that in their natural context feeding on *M. citrifolia* that contains high levels of L-DOPA (Lavista-Llanos *et al.* 2014), *D. sechellia* flies may make CHC profiles more similar to *D. simulans*, lessening the reproductive isolation between these species. Because prior experiments examining aspects of interspecies courtship were conducted in the absence of L-DOPA, future experiments investigating the consequence of L-DOPA on courtship are warranted.

Another gene identified, *Gasp*, resides inside a fine-mapped QTL that explains the greatest amount of variation in octanoic acid resistance between *D. simulans* and *D. sechellia* (Hungate *et al.* 2013). However, this gene was knocked-down with RNAi and shown to not influence OA resistance in *D. melanogaster* functional tests (Andrade López *et al.* 2017) suggesting that it is not the gene that underlies this QTL. Despite the lack of effect on OA resistance, *Gasp* is involved in chitin metabolic processes which may influence other traits associated with host specialization. Interestingly, many other genes with functions in processes associated with the chitin-based cuticle have derived plastic expression responses to L-DOPA in *D. sechellia*, similar to that observed for gene expression responses to OA (Lanno *et al.* 2017) suggesting that *D. sechellia* is altering its cuticle in some way in response to its diet and this may be involved in specializing to eat toxic *M. citrifolia* fruit.

Genes associated with defense against microbial pathogens were also found to be responsive to L-DOPA exposure in this study. Similar to the finding for chitin-related genes, pathogen defense genes are also responsive to OA exposure, possibly suggesting that flies exposed to these chemicals have altered microbial resistance phenotypes. In response to both L-DOPA and OA we observe reduction in the expression of many bacterial defense genes including those involved in bacterial recognition (*e.g.*, *PGRP*s, Supplemental Table 4), as well as many involved in other aspects of immune responses to bacteria (*e.g.*, *edin*, Supplemental Table 4). Because the general trend is toward down-regulation of bacterial defense genes in response to chemicals associated with *M. citrifolia* fruit, our data suggest that flies eating this diet may be immune compromised and therefore more susceptible to bacterial infection when they are exposed to L-DOPA, OA or *M. citrifolia* fruit. Future studies will focus on how the *Drosophila* immune system is altered in these environments with the goal of testing the consequence of food chemical exposure on bacterial infection.

### Knockdown of Esterase 6 alters OA resistance

Our work and that of other research groups have observed L-DOPA effects on various traits in *D. sechellia* (Lavista-Llanos *et al.* 2014) and one of the central aims of this study was to identify candidate genes potentially involved in these derived traits. To do this we focused on the genes that have derived plasticity only observed in *D. sechellia* and found that *Est6* was included in this gene list (Supplementary Table 4). In response to L-DOPA exposure, we found that *Est6* had a significant and marked increase in expression level. This observation was intriguing because our recent study using the chemical synergist tribufos to inactivate all the esterase enzymes simultaneously (Plapp *et al.* 1963; Snoeck *et al.* 2017) found that one or more esterase genes are involved in *D. sechellia* derived resistance to OA (Lanno and Coolon 2019). We therefore knocked down the expression of *Est6* ubiquitously in *D. melanogaster* adults with RNAi and performed OA resistance assays. We found that knock-down of *Est6* resulted in significant decrease in relative survival when individuals were exposed to OA (Figure 5). These experiments were performed in a heterologous host (*D. melanogaster*) based on availability of genetic tools, which is a common approach for tests of gene function, however, confirmation of *Est6* function in OA resistance requires functional tests in *D. sechellia* in future studies. The combination of the OA resistance data and gene expression data collected in this study in conjunction with the prior data showing one or more members of the esterase gene family are involved in OA resistance (Lanno and Coolon 2019), and QTL mapping data showing *Est6* is consistent with regions of the genome contributing to OA resistance all together strongly suggest that *Est6* plays an important role in toxin resistance in *D. sechellia*. Altogether, identification of a derived amino acid substitution fixed in *D. sechellia* that alters a residue that resides in the enzymes active site and was shown to influence *Est6* substrate-specificity and kinetics suggest that both protein coding as well as gene expression changes may contribute to evolutionary changes at this locus.
